# Exploring Bio-Based Polyurethane Adhesives for Eco-Friendly Structural Applications: An Experimental and Numerical Study

**DOI:** 10.3390/polym16172546

**Published:** 2024-09-09

**Authors:** Ana M. S. Couto, Catarina S. P. Borges, Shahin Jalali, Beatriz D. Simões, Eduardo A. S. Marques, Ricardo J. C. Carbas, João C. Bordado, Till Vallée, Lucas F. M. da Silva

**Affiliations:** 1 Departamento de Engenharia Mecânica, Faculdade de Engenharia, Universidade do Porto, Rua Dr. Roberto Frias, 4200-465 Porto, Portugal; 2 Institute of Science and Innovation in Mechanical and Industrial Engineering (INEGI), Rua Dr. Roberto Frias 400, 4200-465 Porto, Portugal; 3 Centro de Recursos Naturais e Ambiente (CERENA), Instituto Superior Técnico, University of Lisbon, 1049-001 Lisbon, Portugal; 4 Fraunhofer Institute for Manufacturing Technology and Advanced Materials (IFAM), Wiener Str. 12, 28359 Bremen, Germany

**Keywords:** automotive industry, adhesive bonding, L-joint, finite element analysis, pine wood, biomaterials

## Abstract

In response to heightened environmental awareness, various industries, including the civil and automotive sector, are contemplating a shift towards the utilization of more sustainable materials. For adhesive bonding, this necessitates the exploration of materials derived from renewable sources, commonly denoted as bio-adhesives. This study focuses on a bio-adhesive L-joint, which is a commonly employed configuration in the automotive sector for creating bonded structural components with significant bending stiffness. In this investigation, the behavior of joints composed of pine wood and bio-based adhesives was studied. Two distinct configurations were studied, differing solely in the fiber orientation of the wood. The research combined experimental testing and finite element modeling to analyze the strength of the joints and determine their failure mode when subjected to tensile loading conditions. The findings indicate that the configuration of the joint plays a crucial role in its overall performance, with one of the solutions demonstrating higher strength. Additionally, a good degree of agreement was observed between the experimental and numerical analyses for one of the configurations, while the consideration of the maximum principal stress failure predictor (MPSFP) proved to accurately predict the location for crack propagation in both configurations.

## 1. Introduction

In recent times, there has been a concerted effort to reduce the impact of fossil-based products, leading to the development and adoption of bio-based materials [1,2,3]. This movement aims to create a more sustainable future by emphasizing the use of tree- and plant-derived products. Specifically, in the construction of eco-friendly load-bearing structures, these materials show great promise, allowing for the creation of composites with natural fibers like flax, jute, and palm. Wood, a timeless resource, has played a significant role throughout history due to its renewable nature [4,5]. Wood, with its multifaceted qualities, stands out as an enticing material choice. Its versatility allows it to take on various shapes, while its impressive durability ensures resilience against wear and environmental factors. The mechanical robustness of wood, combined with its relatively low weight, makes it an appealing option for load-bearing structures. Moreover, its global abundance and economic viability further enhance its allure. What truly sets wood apart is its adaptability. It responds to changing environmental conditions, including temperature fluctuations, varying loading rates, and moisture influences. This adaptability has led to the creation of intricate artifacts with refined geometries a testament to wood’s timeless appeal [6,7]. Wood, as a natural composite material, shares similarities with other composites. However, it does exhibit sensitivity when holes and notches are introduced. Interestingly, traditional joining methods like riveting and bolting are ill suited for wood due to its unique composition [8].

In structural applications, wooden adhesive joints play a pivotal role across diverse industries. In civil engineering, adhesive bonding provides uniform stress distribution without the need for local heating or substrate modifications [2,9]. These joints find use in bridges, buildings, and other infrastructure, enhancing structural integrity and enabling innovative designs. Similarly, in the automotive industry, wood-based adhesive joints offer lightweight and flexible connections, reducing the reliance on mechanical fasteners. Applications include vehicle interiors, panels, and non-structural components. Overall, wooden adhesive joints contribute to sustainable and efficient designs, making them valuable for the future. Bio-based adhesives offer several advantages over traditional petroleum-based counterparts, particularly in terms of sustainability and environmental impact reduction. Derived from renewable resources, these adhesives contribute to a lower carbon footprint by emitting fewer greenhouse gases during production. Additionally, they exhibit lower toxicity to both humans and the environment. In wooden applications, bio-based polyurethane adhesives can be modified for enhanced water resistance and bonding strength [5,10]. These innovative materials pave the way for high-performance alternatives, matching or surpassing synthetic wood adhesives while promoting eco-conscious practices. Adhesively bonded joints offer several advantages over traditional mechanical fasteners (such as bolts, rivets, and welds). First, they distribute stress more uniformly, minimizing stress concentrations. Second, adhesive bonding enhances shock and impact resistance, making joints more robust. Third, adhesives allow effective joining of dissimilar materials, including metals, plastics, wood, ceramics, and more. Fourth, adhesive bonds reduce weight by eliminating the need for additional hardware. Finally, these joints create seamless connections without visible cuts or holes, providing a cleaner overall appearance [11].

In the context of eco-friendly adhesive bonding for wooden structures in industrial applications, various joint configurations are commonly employed. Bio-based adhesives, derived from renewable resources, offer environmental advantages. Among the joint designs, Single-Lap Joints (SLJ) involve overlapping two wooden pieces with adhesive, while Double-Lap Joints (DLJ) provide better load distribution and increased strength. Stepped-Lap Joints distribute stress evenly due to their stepped profile, and Scarf Joints, with tapered overlaps, reduce stress concentration. Additionally, T-joints, where three wooden members intersect, play a crucial role in structural connections. However, the focus lies on L-joints, which are formed when two members meet at a right angle. L-joints are prevalent in wooden frames, furniture, and cabinetry, and their adhesive performance significantly impacts overall structural integrity. The choice of adhesive should consider material properties, load conditions, and environmental factors [12,13,14].

When addressing the numerical simulation of wooden joints, particularly within the context of structural applications, several critical computational aspects come into play. Wood, as a heterogeneous and highly anisotropic material, exhibits ductile behavior under compression but tends toward brittle behavior when subjected to tension or shear forces. To tackle this inherent complexity, researchers have developed advanced 3D constitutive models based on continuum damage mechanics. These models allow for precise representation of wood behavior and have been successfully integrated into finite element frameworks. The validation process, involving embedment and joint tests across different wood species (such as spruce, beech, and azobé), confirms the accuracy of these models by identifying failure modes that align with experimental observations [15,16,17].

Numerical simulations have become indispensable tools in product design and development across various industries, offering substantial cost savings and efficiency improvements [18,19] However, it is worth noting that engineering practices leveraging numerical simulations are not yet widely adopted throughout the entire forest wood chain. Currently, three main approaches characterize the mathematical description of wood material: (1) employing homogeneous volume elements that simplify wood properties and structure [6,20,21,22]; (2) simulating the wood cell wall and structure at a micro-level [23]; and (3) utilizing multiscale modeling techniques [24,25,26]. While numerical methods have found extensive application in civil engineering, particularly in studying deformations under static loads, material failure, and crack propagation using finite element methods (FEM) [27,28,29], the material failure of wood remains an area that has not been thoroughly explored using numerical simulations [30,31]. In the specific case of adhesive joints, cohesive zone elements strike a reasonable balance between calculation time and prediction accuracy.

Numerical models require prior knowledge of material properties. In the context of adhesive joints with biomaterials, the material properties of the substrates and adhesives must be carefully evaluated. Therefore, an experimental investigation must be conducted to characterize these materials. Once the properties are successfully obtained, the numerical model can simulate the overall behavior of the joint. Additional experimental studies within the overall joint must be performed, for validation of the model. Moura and Dourado [17], conducted a study that presented various practical applications with pine wood. These applications encompassed the repair of beams under tensile loading, repair of beams under bending loading, reinforcement of wood structures, steel–wood–steel connections, and wood–wood joints. The researchers utilized typical wood connections and structural details to emphasize the importance of incorporating non-linear fracture mechanics concepts, particularly cohesive zone modeling, in this field. The study achieved a comprehensive qualitative and quantitative representation of the mechanical behavior and failure modes observed in these applications. Hence, its work establishes the proposed procedures as fundamental tools for the efficient design of wood structures. In a separate study, Corte-Real et al., in 2022 [32], investigated single lap joints (SLJs) using different types of wooden substrates. These substrates included natural wood, wood/cork composites, and densified pine wood bonded with a novel polyurethane-based bio-adhesive. It was concluded that the densification process was successful, although it presented an additional challenge due to the resulting surface. Furthermore, increasing the overlap had a positive impact on the energy absorption of the joint, and the addition of cork resulted in a more consistent failure mode and higher strain to failure.

This study focuses on pine wood, a material that is both economically viable and abundantly available. Despite prior research on pine wood, there has been limited exploration of its suitability in L-joint configurations, which are commonly employed in structural applications within the civil and automotive industries. To address this gap, extensive research is essential to comprehend the behavior of adhesively bonded wooden L-joints.

The objective of this research work is to provide design tools for constructing more complex structures using wood. Novel wood-based materials, such as densified wood, hold promise for eventually replacing metals and composites while enhancing sustainability. In this study, the potential of pine wood in L-joint configurations subjected to tension loading conditions was investigated. To achieve this goal, two different joint configurations conducted, an experimental study involving. Additionally, a numerical analysis complemented these experimental efforts.

The findings from this research contribute valuable insights for designing load-bearing structures using pine wood, paving the way for eco-friendly alternatives in various industries.

## 2. Materials and Methods

In this section, a careful description of the materials used, including their properties and geometry and dimensions of the joint are provided. Additionally, the testing conditions of all experiments are revealed to ensure transparency and reproducibility of the results. Finally, this section presents the conclusive findings obtained from the experiments.

### 2.1. Materials

#### 2.1.1. Substrate

In this study, pine wood served as the primary substrate, sourced from the southern regions of Portugal. The selection was based on its cost-effectiveness, widespread availability, and suitable mechanical properties. Notably, the mechanical behavior of wood is intricate and highly dependent on various factors, including species, growth conditions, grain slope and size, defects, knots, shakes, and age. For bio-substrate applications, beams exhibiting maximum symmetry and straight grains were chosen, while the use of knotted timber was avoided. The mechanical and cohesive properties of pine wood were experimentally determined by Moura et al. [21] and are presented in Table 1, Table 2 and Table 3.

**Figure 1:**
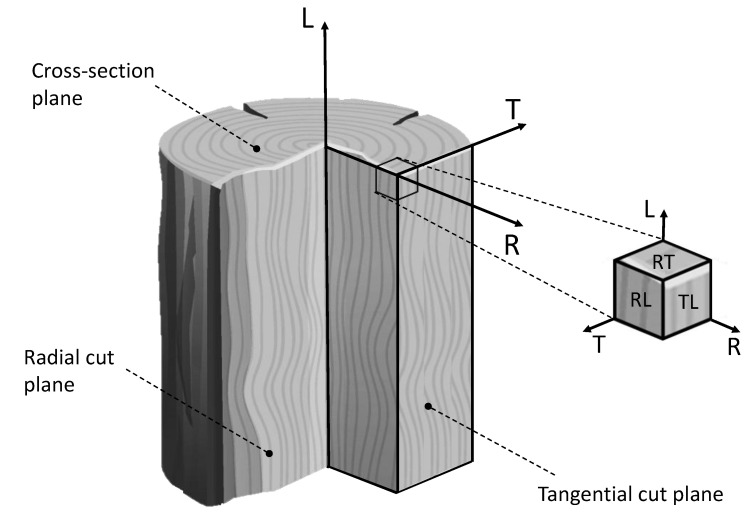
Main orthotropic directions and planes in wood.

The strength and fracture characteristics of wood significantly depend on the direction of loading. Neglecting this consideration could lead to severe wood failure. Wood cells align with the grain direction, resulting in maximum strength when the load is applied along this axis. To describe wood’s mechanical and physical properties, a cylindrical coordinate system—comprising longitudinal (L), radial (R), and tangential (T) directions—is commonly employed due to the circular grain orientation (see Figure 1).

The moisture content of the wood fell within the typical range of 8% to 25% by weight, suitable for various wood types and applications. It remained dry, with a moisture content not exceeding 19%, which aligns with the limit for sawn lumber design. This moisture level significantly impacts the wood’s properties, including dimensional stability, strength, durability, and resistance to biological factors.

For a better understanding of the different planes considered in this study, Figure 1 presents the main orthotropic directions and planes in wood.

#### 2.1.2. Bio-Adhesive

A bio-based moisture-curing polyurethane, composed of 70% natural materials, was employed to bond the substrates. As a moisture-curing adhesive, it reacts with the moisture present in the substrate or the surrounding air during the curing process. To achieve a flawless bond without voids or defects, the adhesive must directly contact the substrates in a zero-thickness condition, allowing it to interact with the hydroxyl (OH) groups in the wood. This interaction exploits both mechanical interlocking and chemical bonding with the wooden substrates. These chemical bonds form between the OH groups in the wood and key components of the adhesive, synergistically enhancing the overall adhesive strength. Notably, the moisture content of the wood significantly influences the curing process of this bio-adhesive. Maintaining a consistent moisture level is crucial for determining the adhesive’s curing time. Therefore, all substrates used in this study adhered to a specified moisture content range of 12–20%, meticulously ensured by the wood supplier. This precise control of moisture content ensures uniformity in the adhesive’s curing process, ultimately enhancing its performance and reliability. The adhesive cures at 100 °C for a duration of 8 h. It is essential to mention that this bio-adhesive is currently in the prototype stage and has not been commercially released; however, it shows potential as a sustainable alternative to synthetic adhesives. It is produced in an irreversible reaction, without humidity, in a reactor under a nitrogen atmosphere and heating is performed with a thermal oil coil. It uses an aliphatic isocyanate as a basis, which contains 70% plant matter, and thus is more easily biodegradable. Manufacturing the bio-adhesive is estimated to consume 15 to 20% less energy than those derived from petroleum. The adhesive was developed by Professor João Bordado from Instituto Superior Técnico and was specifically formulated to display excellent compatibility and adhesion to wood substrates.

The experimental characterization of the adhesive properties was conducted by Jalali et al. [33]. In terms of mechanical characterization, testing using a modified thick adherend shear test (TAST) specimen revealed a minimum shear strength value of 12.4 MPa. The author mentions tests utilizing modified double-cantilever beam (DCB) and end-notched flexure (ENF) specimens provided *G_Ic_* and *G_IIc_* values of 0.16 N/mm. In butt joints, the tensile strength was measured to be 16.5 MPa.

### 2.2. Joint Manufacturing

**Figure 2:**
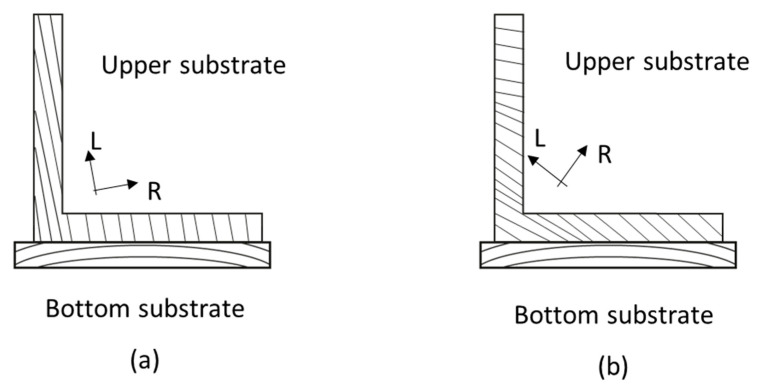
Illustration of the cross-section dimensions of the joints: (**a**) vertical orientation of the upper substrate and (**b**) inclined orientation of the upper substrate.

The joint assembly comprises an L-shaped upper substrate and a flat bottom substrate. When manufacturing joints using orthotropic wood, one critical parameter is the orientation of the wood rings. Depending on the substrate, various orientations were deliberately considered. For the bottom substrate, the preferred ring alignment is depicted in Figure 2. However, achieving flawless symmetry around a centrally aligned vertical axis was not consistently feasible. As for the upper substrate, two distinct configurations were studied: a nominally vertical orientation (a) and an inclined one (b). Although the term ‘vertical’ simplifies the description, it is important to note that the actual orientation may not be perfectly vertical. By observing both orientations in Figure 2, it can be understand the differences in upper substrate alignment.

**Figure 3:**
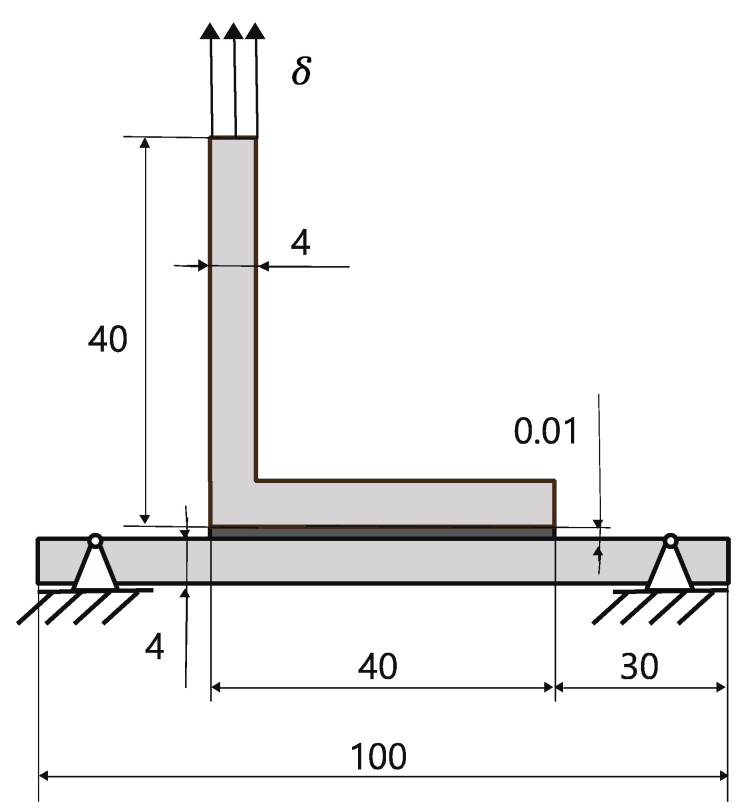
Dimensions of the L-joints, in mm, and the direction of the displacement, δ.

The manufacturing process involved preparing the substrates, which had previously been conditioned at 23 °C and 50% rH. To ensure a similar topography of the substrates, all specimens were cut in the same equipment and from wood extracted from the same batch. This was followed simply by air blasting to remove all dust from the surface. This process produced surfaces that were consistent in their surface roughness. A thin layer of adhesive was evenly spread across the overlap region on both substrates. The substrates were then carefully aligned and firmly pressed together. To ensure a robust bond, pressure was exerted on the overlap area using a plate and clamps. The assembled substrates were subsequently positioned in an oven to ensure the curing of the adhesive. Finally, the joints were removed, and any surplus adhesive along the edges was cleaned as needed. Figure 3 illustrates the final assembly and provides an overview of the joint dimensions. After the assembly, all samples were again conditioned at 23 °C and 50% rH.

### 2.3. Testing Conditions

**Figure 4:**
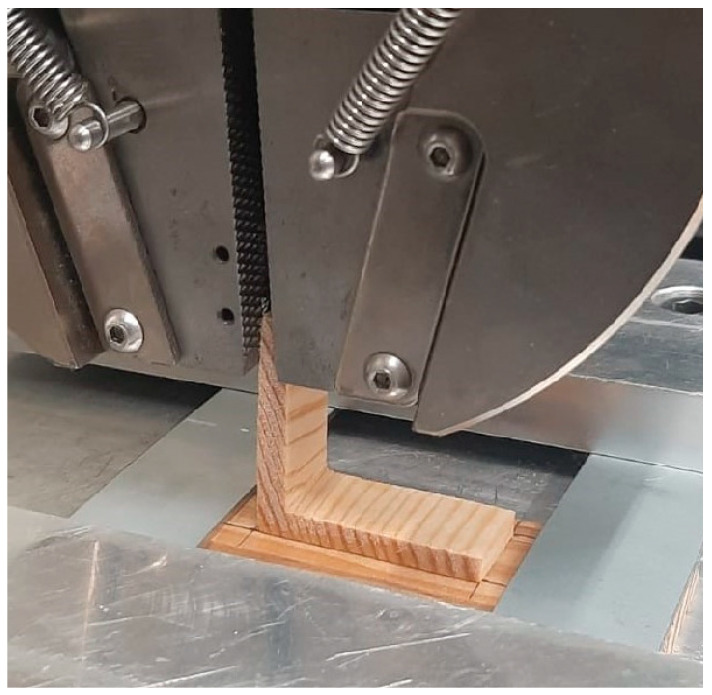
Experimental setup for testing the L-joints.

The L-joints manufactured for this work were tested via the application of a tensile load on the upper substrate and were conducted by the quasi-static test machine. The tensile tests were performed using a universal testing machine INSTRON 3367^®^ (Illinois Tool Works, Hopkinton, MA, USA) with a load cell capacity of 30 kN, and both the load, *P*, and displacement, *δ*, were recorded by the machine at a constant rate of 1 mm/min. Figure 3 and Figure 4 show the dimensions of the specimens and the setup utilized to conduct the testing procedures. Furthermore, the width of upper and bottom substrate was 15 and 25 mm, respectively. The clamped length was controlled during the tests, ensuring that it was always set to be 15 mm. It should be noted that at least five specimens were tested for each condition. The tests were conducted in laboratory conditions of temperature and humidity.

### 2.4. Numerical Details

A finite element model was developed to simulate the L-joints in the attempt to predict the real behavior of the structure. The Abaqus 2022^®^ software was used to carry out the analysis. Numerical models for the two previously mentioned configurations (Figure 2) were made considering the mechanical properties previously reported for the pine wood and the bio-adhesive.

**Figure 5:**
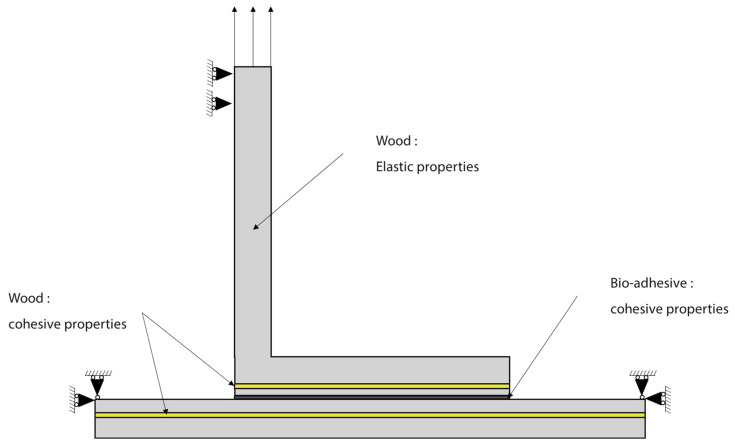
Boundary conditions and cohesive elements used in the numerical model of the L-joint.

To model the adhesive layer, cohesive elements were utilized, and their behavior was defined by a triangular traction-separation law. The properties of this law, including strength and fracture properties of the novel bio-adhesive, were determined by Jalali et al. [33]. For the wood substrates, a homogenous orthotropic layer was employed with the corresponding elastic properties presented in Table 1, Table 2 and Table 3. Additionally, a layer of cohesive elements was positioned at a 0.15 mm distance of the interface of both upper and bottom substrates to allow for possible delamination of the wood. This distance was selected according to experimental observation, which has shown that this type of failure occurs one or two fibers away from the surface. This usually corresponds to a distance between 0.1 and 0.2 mm from the surface. The triangular cohesive law governing the elements in the wood was defined based on the strength and fracture properties of natural pine wood, as determined through experimental analysis by Moura and Dourado [17]. A visual representation of the model can be seen in Figure 5.

**Figure 6:**
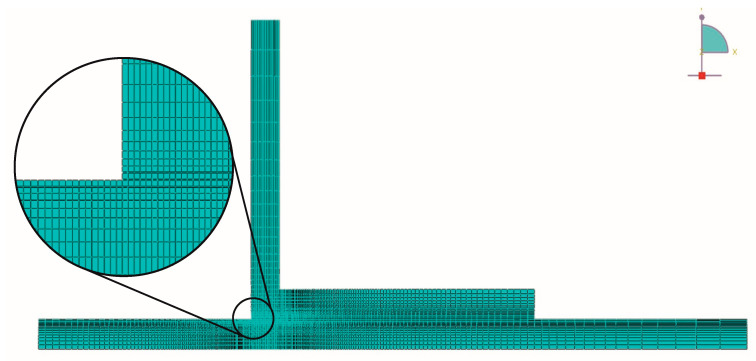
Mesh applied to the L-joint.

In this study, we implemented boundary conditions and mesh configurations meticulously to ensure precise finite element simulations. First, we fixed the horizontal and vertical directions of the two upper edges of the bottom substrate. Simultaneously, the upper edge of the upper substrate experienced vertical upward displacement while remaining horizontally constrained. To achieve reliable results, we employed a high mesh density, comprising 11,740 four-node bilinear plane strain quadrilateral elements (CPE4) and 700 four-node two-dimensional cohesive elements (COH2D4), as depicted in Figure 6. Additionally, we discretized the substrate thickness using over 20 elements to capture bending phenomena accurately. Notably, a mesh refinement strategy was applied to account for localized stress effects in the adhesive region. These considerations collectively enhance the accuracy and reliability of our FEM simulations.

## 3. Experimental and Numerical Analysis

### 3.1. Strength Analysis

**Figure 7:**
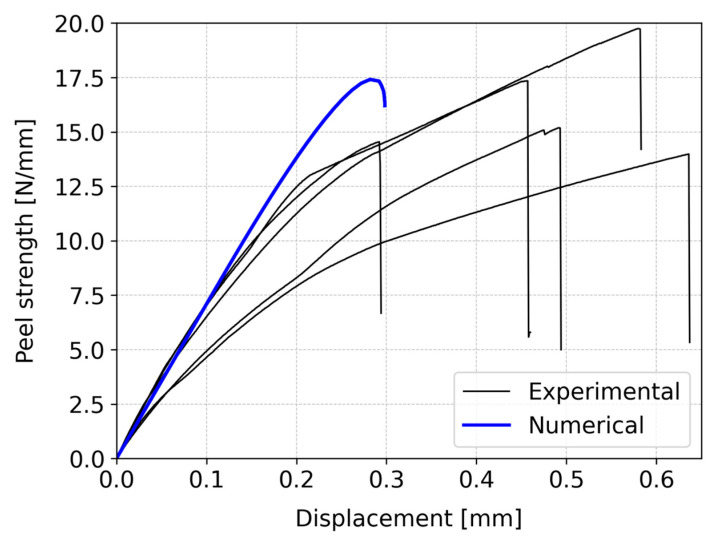
Comparison between experimental and numerical peel strength—displacement curves for the vertical configuration of the L-joint.

**Figure 8:**
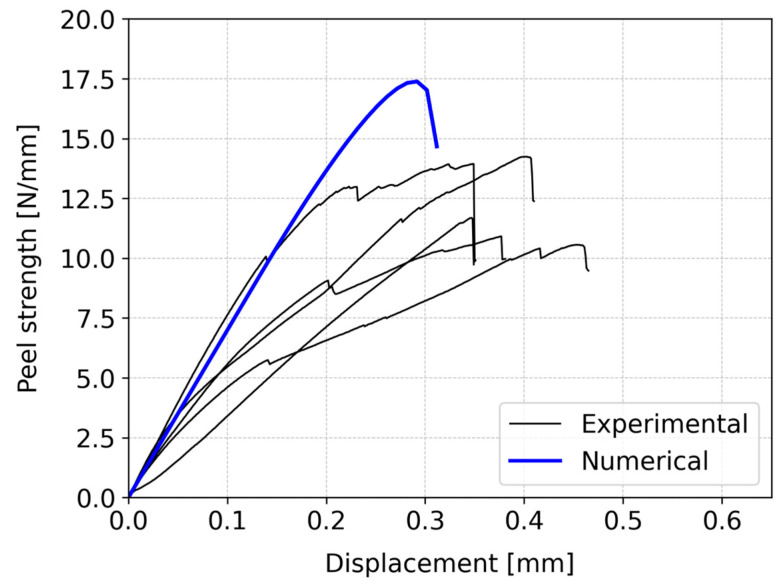
Comparison between experimental and numerical peel strength—displacement curves for the inclined configuration of the L-joint.

A strength analysis was conducted considering both configurations. The peel strength—displacement curves were derived from experimental procedures, directly extracted from the testing machine. The load retrieved was divided by the upper substrate width in order to determine the strength of each tested specimen. It is important to highlight that, to eliminate the influence of machine component measurements, the displacement values for the experimental curves were adjusted using a correction factor of 0.6. This value was experimentally determined and represents an adjustment of the compliance of the testing machine and the used test setup. Figure 7 and Figure 8 present the results for the vertical and inclined configurations, respectively.

In the case of pine wood with a vertical fiber orientation of the upper substrate (Figure 7), the average peel strength of the joint was 16.2 ± 2.1 N/mm, with a corresponding displacement of 0.49 ± 0.12 mm. On the other hand, for the alternative geometry (Figure 8), the joint sustained an average peel strength of 12.2 ± 1.5 N/mm, resulting in a displacement of 0.38 ± 0.03 mm. The energy absorbed by the joints was determined by the area under the peel strength—displacement curves. The energy absorbed up to the point of failure for the vertical configuration was approximately 2.6 higher than the absorbed energy by the inclined configuration.

A comparison between these two cases reveals notable differences. Specifically, the first case exhibited approximately 25% higher peel strength than the second case, along with a superior displacement-to-failure capacity of around 22%. Furthermore, the observed behavior in the two cases significantly differs. In the vertical configuration (Figure 7), there is a noticeable shift in the material’s behavior, marked by a distinct change in the slope of the curve. Initially, the material demonstrates elastic behavior, but it subsequently transitions to a plastic behavior, allowing for more displacement before failure occurs. On the other hand, in the inclined configuration (Figure 8), cracks in the wood are present, which were not observed in the vertical configuration. During the testing, the propagation of cracks could be heard, although they would not be visible. This indicated the presence and propagation of microcracks during testing, which would influence the fracture behavior of this particular configuration. These microcracks suggest the initiation of localized damage in the inclined configuration, contributing to its unique behavior. In conclusion, the two configurations not only vary in peel strength and displacement capacity but also exhibit distinct behaviors during testing.

Moreover, it is crucial to emphasize that the vertical configuration demonstrates superior characteristics including higher energy absorption, greater peel strength, and increased displacement capability prior to failure. The presence of vertical fiber orientations in the overlap region results in the loading being applied along the strongest direction, thereby enhancing the stiffness and peel strength of the joint. The vertical alignment of the fibers maximizes their load-bearing capacity, leading to improved overall performance of the joint.

The numerical computations conducted for both configurations are presented in form of resulting peel strength—displacement curves. These results were directly extracted and compared to the experimental data. Figure 7 illustrates the comparison between numerical and experimental results for the vertical configuration, while Figure 8 presents the corresponding comparison for the inclined configuration.

In the case of the vertical configuration, the numerical analysis simulated the peel strength of 17.4 N/mm with a displacement of 0.28 mm. In contrast, the experimental peel strength was reported as 16.2 ± 2.1 N/mm, accompanied by a displacement of 0.49 ± 0.12 mm. This indicates that the numerical model presents a reasonable agreement for the failure load. However, there is some deviation in displacement between the numerical and experimental results. Specifically, up to approximately 0.2 mm, the numerical and experimental results align well. Beyond this point, a noticeable divergence emerges in the behavior of the two sets of results. One possible explanation for this disparity is that the experimental data suggests signs of plastic behavior, allowing for greater displacement before failure. Unfortunately, the numerical simulation did not account for this phenomenon due to the lack of information to simulate such behavior. Despite the limitation of considering only the elastic behavior of wood, the numerical results still offer reasonably accurate predictions.

In the inclined configuration, the numerical analysis determined peel strength of 17.3 N/mm with a displacement of 0.29 mm. In contrast, experimental testing yielded a peel strength of 12.2 ± 1.5 N/mm, accompanied by a displacement of 0.38 ± 0.03 mm. As mentioned, in this configuration, the presence of microcracks in the experimental testing was noted, contributing to the observed discrepancy, since they are not considered in the finite element analysis (FEA). Consequently, the FEA tends to predict higher failure loads compared to the experimental results, proving to have a worse performance for this configuration in terms of the failure load. However, a smaller discrepancy in the final displacement between numerical and experimental data was observed when compared to the previous configuration.

Comparing both numerical results shows strikingly similar patterns in terms of the failure load, displacement, and the peel strength—displacement curve shape. However, real-world behavior diverges. The experimental data are scattered, and the authors believe that the source of these dispersion is mainly caused by the wood itself and thus a key limitation associated with using these materials. Even when sourced from the same specimen, the different locations along the wood can have drastically varied behavior. In a design-based approach, one must find ways to avoid having to precisely characterize these geometrical and material uncertainties and try to employ a more general approach. In the vertical setup, the numerical and experimental results closely match on the failure load, though not on the displacement. The numerical model does not consider plasticity, unlike the experiments, which show more displacement tolerance. In the inclined case, discrepancies arise due to microcracks unaccounted for in the numerical analysis. Despite these differences, the numerical model remains a valuable tool for predicting joint strength, particularly within the elastic range.

### 3.2. Failure Mode

**Figure 9:**
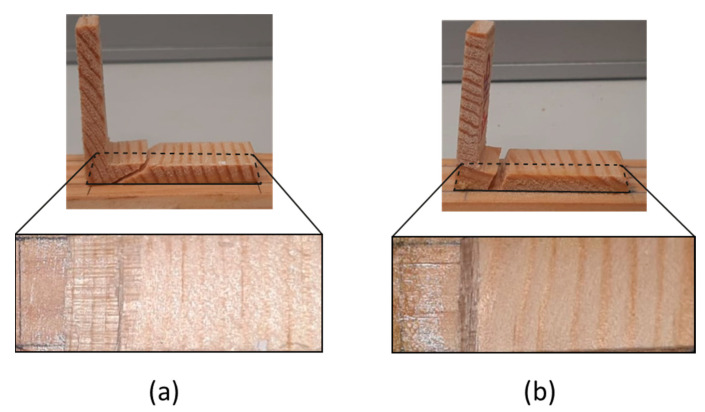
Failure mode observation for (**a**) vertical configuration and (**b**) inclined configuration.

Following the testing phase, a detailed analysis was conducted to identify the type of failure modes observed for both cases. Two types of behavior were registered. In both cases, a visibly non-reflective surface in the overlap region strongly suggests the occurrence of delamination. In the case of the vertical configuration, the delamination process involved a greater number of fibers being pulled, accompanied by a fracture occurring within the wood at a very oblique angle (Figure 9a). Conversely, for the inclined configuration, the plane of failure exhibited a more vertical orientation and was sudden, resulting in a flatter failure surface (Figure 9b). All of the tested specimens failed as presented in Figure 9.

**Figure 10:**
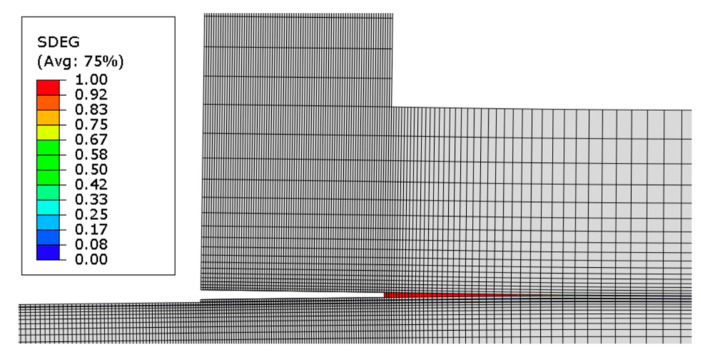
Damage analysis (SDEG) for L-joint at failure.

In the numerical analysis, delamination was predicted as the predominant failure mode, and it was imperative to understand this phenomenon. The monitoring of damage progression utilized the state variable SDEG (Figure 10), which corresponds to stiffness degradation of the cohesive element, with zero indicating the absence of damage, and a linear increase in SDEG signifying damage progression until reaching one, resulting in the failure of the cohesive element.

**Figure 11:**
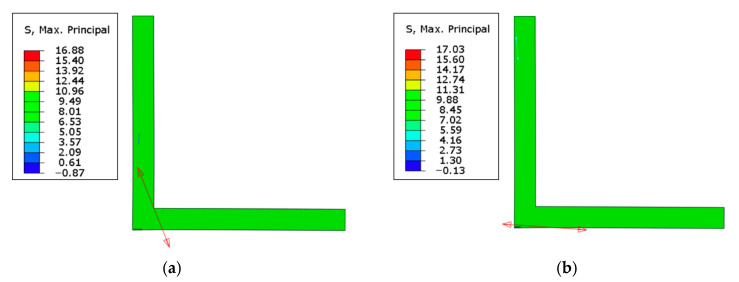
Maximum principal stress distribution in the FEA: (**a**) vertical configuration and (**b**) inclined configuration, where the red arrows indicate the local direction where the maximum principal stress was attained.

To address the discrepancies observed between the numerical model and experimental observations, and considering a global approach to the problem, a thorough analysis was conducted, leading to the consideration of the maximum principal stress criteria, specifically the maximum principal stress failure predictor (MPSFP). According to MPSFP, a fracture occurs when the local strength is exceeded by the maximum principal stress in a multiaxial stress system. Using this approach, it was possible to check, for a given displacement, the plane in which the maximum principal stress reached a value greater than the material’s strength. As shown in Figure 11, although in general the specimen is below the strength of the RL plane of 16 MPa, the red arrow indicates that locally, in this plane, this value is exceeded, with 17 MPa and the orientation represented by the same arrow. Since a crack propagates in the direction perpendicular to the plane with the maximum principal stress, this numerical simulation allows us to obtain the correct orientation of the crack propagation when compared to the experimental results. Comparing the images in Figure 9 with the results in Figure 11, a good agreement can be observed.

## 4. Conclusions

This study focused on investigating L-joints for the automotive industry by incorporating biomaterials, specifically pine wooden substrates with a novel polyurethane-based bio-adhesive. Two different configurations using pine wood were analyzed through strength analysis and failure mode. This study involved experimental and numerical analyses, which were compared to each other.

In comparing the two configurations, significant distinctions emerged. The first configuration demonstrated a notable 25% increase in strength and a 22% improvement in displacement-to-failure capacity compared to the second. These differences were accompanied by varying material behaviors during testing. In the vertical setup, the material initially exhibited elastic behavior but shifted to plastic behavior, allowing for more displacement before failure. Conversely, the inclined configuration indicated the presence of microcracks, which suggests a localized damage not observed in the vertical setup. These findings highlight differences not only in strength and displacement capacity but also in observed behaviors. The vertical configuration displayed superior characteristics, including higher energy absorption, increased strength, and enhanced displacement capability before failure. This superiority stemmed from vertical fiber orientations in the overlap region, facilitating loading along the material’s strongest direction. The analysis of failure modes identified delamination as the predominant issue in both cases, with visible non-reflective surfaces indicating this phenomenon. In the vertical configuration, delamination involved more fibers being pulled, resulting in an oblique fracture within the wood. In contrast, the inclined configuration exhibited a more vertically oriented failure, leading to a flatter failure surface.

Regarding numerical versus experimental results, the numerical analysis presented a reasonable agreement with experimental data in terms of the failure load for the vertical configuration. However, deviations in displacement were observed, primarily due to the unaccounted plastic behavior in the numerical simulations. Regarding the inclined configuration, during the testing of the inclined configuration, the propagation of cracks could be heard, although they were not visible. Thus, the authors consider that the propagation of microcracks during testing was an influencing parameter in the fracture behavior of this particular configuration, contributing to the different behavior observed in the numerical model.

To address these discrepancies, this study introduced the maximum principal stress criteria, specifically the maximum principal stress failure predictor (MPSFP). MPSFP stipulates that fracture occurs when the maximum principal stress surpasses local strength in a multiaxial stress system. The verification revealed that, at a specific displacement, the maximum principal stress exceeded local strength for the RL plane, explaining the observed crack propagation. Although there was an attempt to use a simple model, which is able to reproduce the behavior of the wood without the need for highly complex characterization procedures, in future works, the present study should be expanded to create a more accurate model for the bonded wood. Furthermore, it will be of great interest to use these same models to study and predict the behavior of these structures considering the effects of aging and dynamic loads. In fact, one of the main problems facing these wooden structures is that their long-term behavior is not yet fully understood or modeled.

In summary, this study underscores differences in behavior between configurations, acknowledging the reliability of numerical modeling while recognizing limitations related to plasticity and microcracks. It also emphasizes the significance of considering failure modes and criteria for accurate simulations.

## Figures and Tables

**Table 1:** Nominal elastic properties of Pinus pinaster. (Young’s modulus (*E*), Poisson’s ratio (*ν*), and shear modulus (*G*)) [17].

*E_L_* [GPa]	*E_R_* [GPa]	*E_T_* [GPa]	*υ_TL_*	*υ_RL_*	*υ_RT_*	*G_RL_* [GPa]	*G_TL_* [GPa]	*G_RT_* [GPa]
12.0	1.91	1.01	0.51	0.47	0.31	1.12	1.04	0.29

**Table 2:** Nominal strength properties of Pinus pinaster [17].

*σ_uL_* [MPa]	*σ_uR_* [MPa]	*σ_uT_* [MPa]	*σ_uRL_* [MPa]	*σ_uTL_* [MPa]	*σ_uRT_* [MPa]
97.5	7.90	4.20	16.0	16.0	4.50

**Table 3:** Cohesive parameters of Pinus pinaster in the RL fracture system [17].

*G_Ic_* [N/mm]	*G_IIc_* [N/mm]	*σ*_1*,I*_ [MPa]	*σ*_1*,II*_ [MPa]
0.264	0.94	5.34	9.27

## Data Availability

The raw/processed data required to reproduce these findings cannot be shared at this time as the data are part of an ongoing study.
